# Secondary Structures of Proteins: A Comparison of Models and Experimental Results

**DOI:** 10.1021/acs.jproteome.0c00986

**Published:** 2021-02-23

**Authors:** Mónika Bokor, Ágnes Tantos

**Affiliations:** † Institute for Solid State Physics and Optics, Wigner Research Centre for Physics, Konkoly-Thege út 29-33, 1121 Budapest, Hungary; ‡ Institute of Enzymology, Research Centre for Natural Sciences, Magyar Tudósok Körútja 2, 1117 Budapest, Hungary

## Abstract

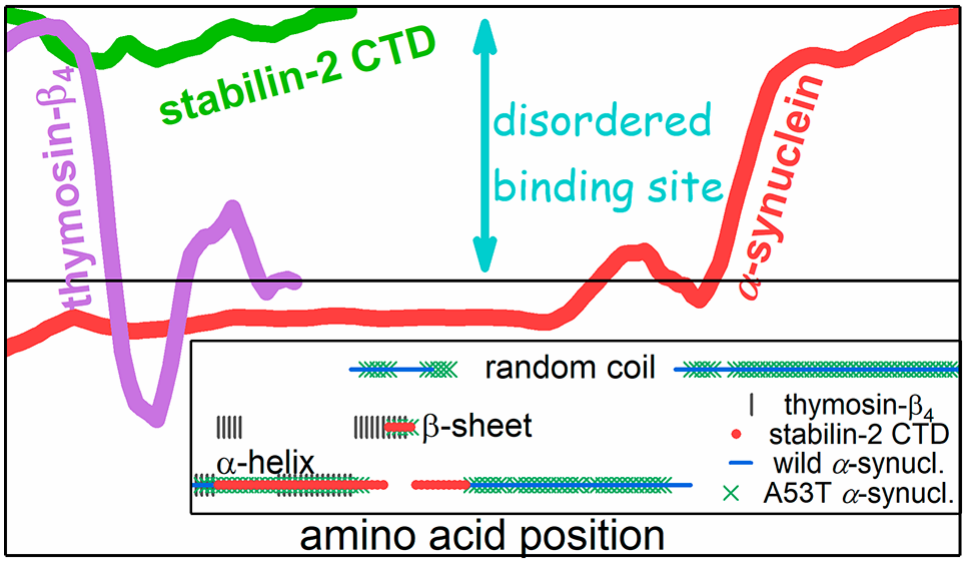

Secondary structure predictions of proteins were compared to experimental results by wide-line ^1^H NMR. IUPred2A was used to generate predictions of disordered protein or binding regions. Thymosin-β_4_ and the stabilin-2 cytoplasmic domain were found to be mainly disordered, in agreement with the experimental results. α-Synuclein variants were predicted to be disordered, as in the experiments, but the A53T mutant showed less predicted disorder, in contrast with the wide-line ^1^H NMR result. A disordered binding site was found for thymosin-β_4_, whereas the stabilin-2 cytoplasmic domain was indicated as such in its entire length. The last third of the α-synuclein variant’s sequence was a disordered binding site. Thymosin-β_4_ and the stabilin-2 cytoplasmic domain contained only coils and helices according to five secondary structure prediction methods (SPIDER3-SPOT-1D, PSRSM, MUFold-SSW, Porter 5, and RaptorX). β-Sheets are present in α-synucleins, and they extend to more amino acid residues in the A53T mutant according to the predictions. The latter is verified by experiments. The comparison of the predictions with the experiments suggests that helical parts are buried.

## Introduction

New recently developed and older sequence-based predictors are widely applied for the characterization and prediction of protein structure and function. Several accurate predictors have been produced, many of which are based on machine-learning models and evolutionary information generated from multiple sequence alignments. Here the particular predicted protein secondary structures (SSs) are compared with the structural information gained by wide-line nuclear magnetic resonance (NMR) experiments for verification. To get a more reliable prediction, several prediction methods were applied, and the results were averaged.

Two protein systems were investigated, both of which are of medical importance. Thymosin-β_4_ (Tb4) and the stabilin-2 cytoplasmic domain (CTD) constitute one such system, as a 1:1 complex has a major role in apoptotic cell clearance.^1^ Wild type (WT) and A53T α-synucleins (α-Ss) are the second system, which is involved in Parkinson’s disease.^2^ The A53T mutation in α-synuclein is related to autosomal-dominant early onset familial Parkinson’s disease.^3^ All of these proteins are intrinsically disordered proteins (IDPs)^4,5^ that have no single well-defined tertiary structure under native conditions.

Wide-line ^1^H NMR experimental results provide unique information on the interactions of proteins with the solvent water in the form of a melting diagram (MD).^4−6^ The MDs (the amount of mobile hydration water measured by wide-line NMR versus the temperature/potential barrier; see the Supporting Information) contain experimental information on structural properties of the studied proteins.^5−8^ A constant section of MDs at low temperatures/potential barriers is a sign of ordered protein regions, that is, secondary structural elements. A constantly increasing amount of mobile hydration water at higher temperatures/potential barriers reflects heterogeneous water–protein interactions, which are consequences of the disordered protein structure. The HeR parameter of MDs serves as a ratio of the heterogeneous/disordered protein regions of the solvent-accessible surface (SAS), the complementary of which is the ratio of the secondary structural elements. HeR is measured from MDs as a ratio of the thermal width of the heterogeneous behavior to the thermal distance of the mobile hydration water appearance. Both are measured from 0 °C.

In the following work, we compare SS predictions with wide-line ^1^H NMR experimental results and evaluate the predictions based on their agreement.

## Results and Discussion

**Figure 1:**
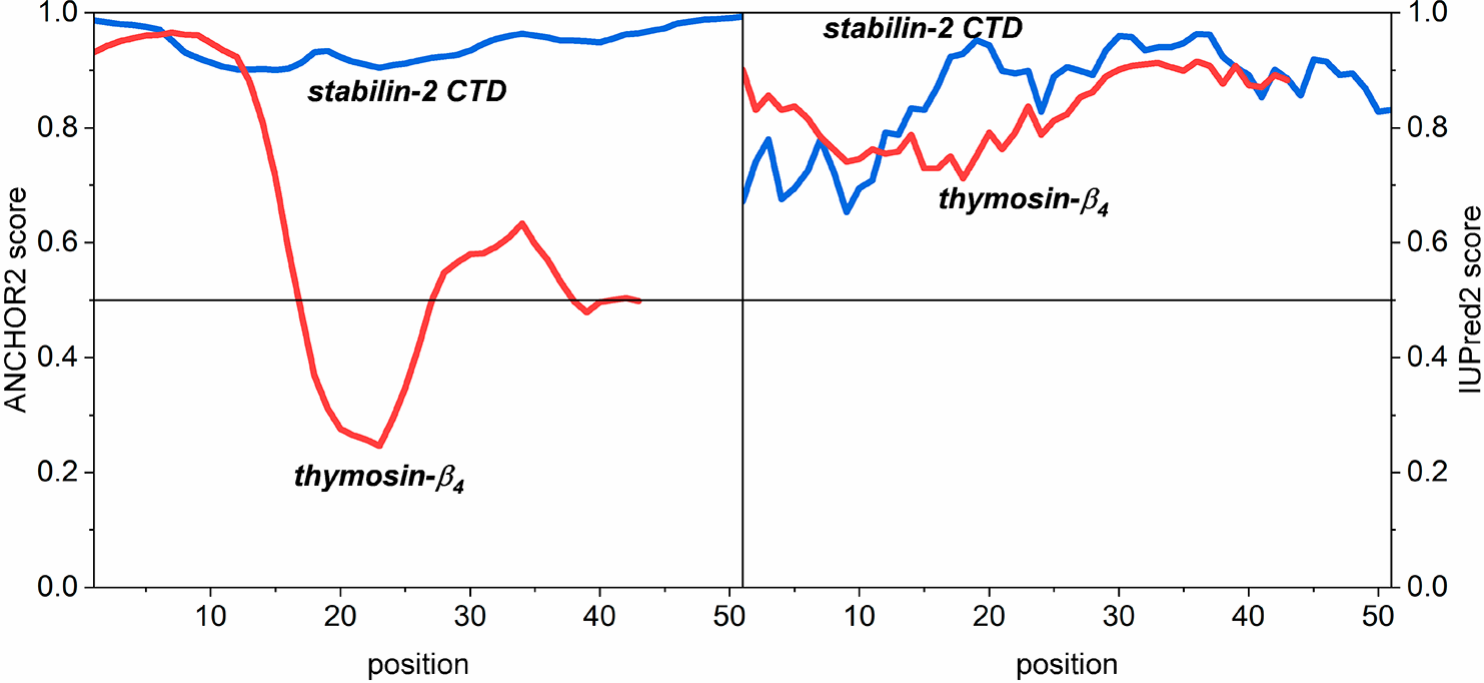
Prediction of protein disorder and disordered binding sites^9^ for thymosin-β_4_ (red) and the stabilin-2 CTD (blue) by ANCHOR2 (left) and IUPred2 (right) programs.

Calculations to establish the presence of disordered protein regions and disordered binding regions were made for Tb4 and the stabilin-2 CTD by IUPred2 and ANCHOR2, respectively (Figure 1). IUPred2 showed that the degree of disorder for the whole Tb4 sequence is 83(1)%. The degree of disorder is 71(1)% for the first 11 amino acid residues and 90.7(6)% for the last 35 amino acid residues of the stabilin-2 CTD. The Stabilin-2 CTD is disordered to a high degree in its whole length. HeR, the ratio of heterogeneously binding interface,^7^ established that individual Tb4 and stabilin-2 CTD have very heterogeneous bonds with mobile hydration water molecules. According to IUPred2 predictions, they are highly disordered in their entire length, in accordance with experimental wide-line ^1^H NMR results.^4^

ANCHOR2, which recognizes disordered binding regions with score values >0.5, shows a definite binding region at the N-terminus of Tb4 and a less expressed one in the second half of the protein. Residues 1–16 and 28–37 are considered as binding sites in Tb4. However, ANCHOR2 signifies the whole stabilin-2 CTD as a binding region.

**Figure 2:**
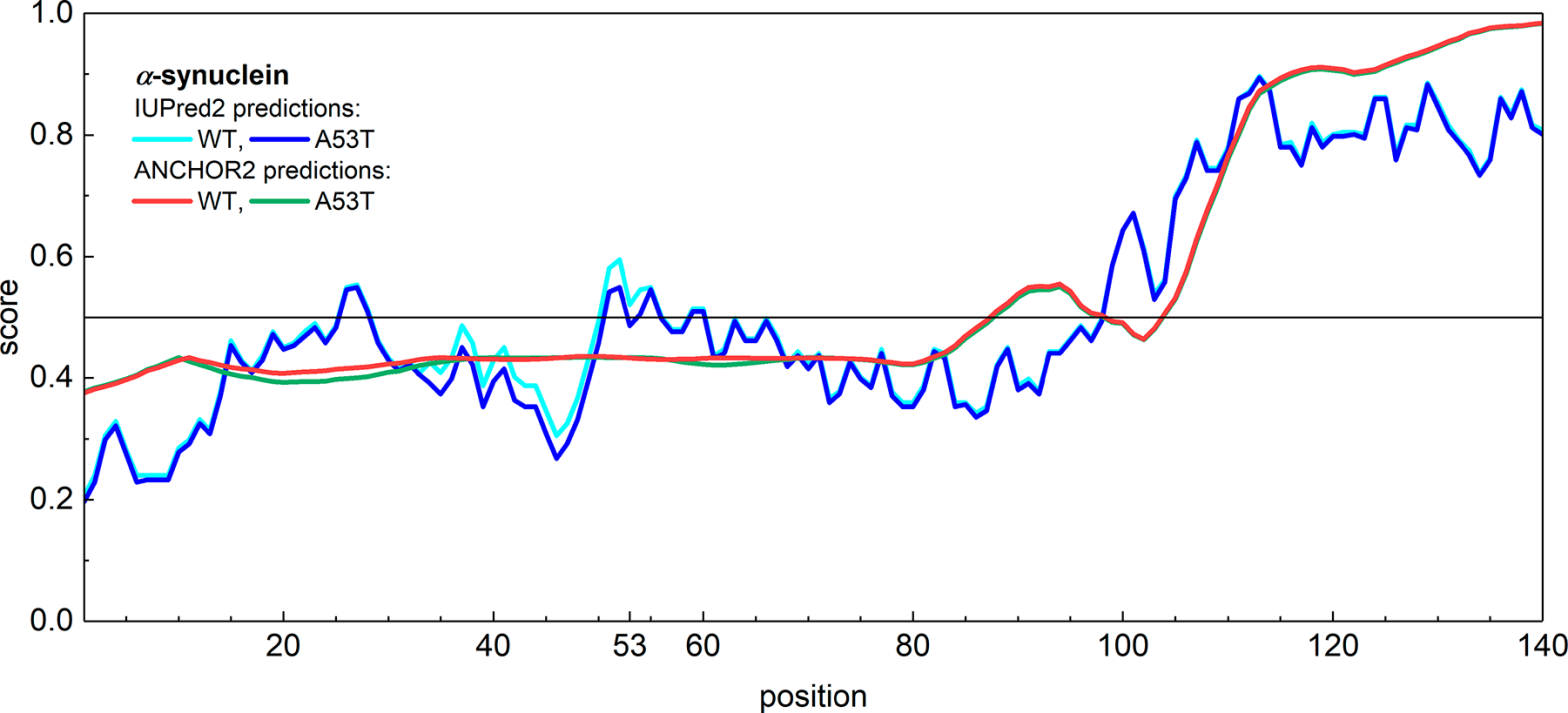
Prediction of protein disorder and disordered binding sites^9^ for wild-type and A53T α-synuclein by the IUPred2 and ANCHOR2 programs.

Predictions with IUPred2A were performed for wild-type (WT) and A53T mutant α-Ss (Figure 2). IUPred2 predicted on the basis of sequence that two-thirds of their N-termini have a 42(8)% degree of disorder. The A53T mutant was predicted to be a little more ordered, with a more pronounced difference around and before the site of mutation at residues 34–54 (Figure 2). ANCHOR2 gives identical predictions for the two variants of α-Ss. The first 80–100 residues do not form a disordered binding site (average score of 0.42(8)), but the last 30 residues at the C terminus do form a disordered binding site (score of 0.82(4)). Residues 100–110 form a transitional region between the two states.

These results agree with the fact that the α-Ss are intrinsically disordered, as seen by wide-line ^1^H NMR.^5,10^ More precisely, 68(4)% of their SAS is heterogeneous/disordered.

The β-sheet formation increases near the site of mutation in the N-terminal region^11^ due to the amino acid change; that is, the A53T mutation causes slightly more order around the mutation site. This experimental result verifies the prediction that at residues 34–54, the mutant is more compact than the wild-type α-S. Wide-line ^1^H NMR experiments, on the contrary, indicate that there is more mobile hydration water at the heterogeneously hydrated regions, which means a more open structure.^5,10^

The SSs of Tb4 and the stabilin-2 CTD were predicted by 3-state and 8-state methods. Both methods provided the same results, although the 8-state methods are less accurate than the 3-state methods.

**Figure 3:**
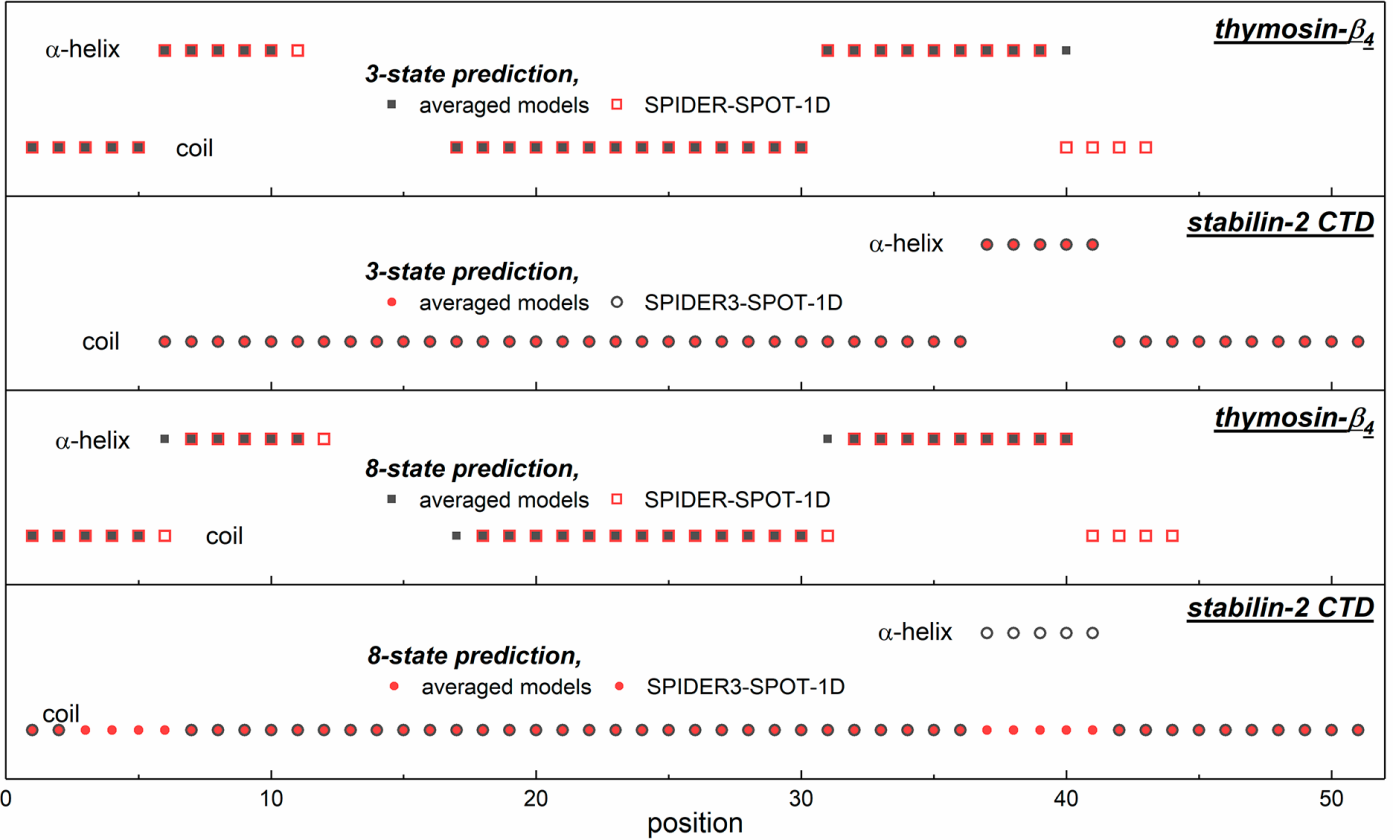
Predicted 3- and 8-state secondary structures for thymosin-β_4_ and the stabilin-2 CTD. The average structure of the five modeling programs (SPIDER3-SPOT-1D, PSRSM, MUFold-SSW, Porter 5, and RaptorX) and that of the separate SPIDER3-SPOT-1D prediction are shown.

The 3-state SS prediction methods (SPIDER3-SPOT-1D, PSRSM, MUFold-SSW, Porter 5, and RaptorX) resulted in a structure for Tb4 containing only coils and helices (Figure 3). Helices are predicted to be at the N and C terminal ends of the sequence. They extend to 12 and 23% of the Tb4 length, respectively. More precisely, the first helix is formed by residues 6–10 in the average predicted SS or by residues 6–11 in the SPIDER3-SPOT-1D predicted SS at the N-terminal end, and the second helix is formed by residues 31–40 or 31–39, respectively, at the C-terminal end in these predictions. The second helix is present according to each of the five methods. On average, the second helix is longer by one residue, and it is predicted to be only nine residues long by the SPIDER3-SPOT-1D method. The motifs run to 11.6, 14.0, 23.3, and 20.9% one after another. This prediction is in good agreement with the solution NMR structures of the free and actin-bound Tb_4_.^12^

The 8-state prediction models result in almost the same SS for Tb4 compared to the 3-state models (Figure 3), with only very small differences. The 23% helix length fit the HeR value measured by wide-line ^1^H NMR very well, according to which Tb4 contains 22(1)% secondary structural elements.^4^ The shorter helix is stabilized by the binding to actin monomers and is highly flexible in solution;^12^ therefore, it is not visible for wide-line ^1^H NMR.

The stabilin-2 CTD has a 17(3)% ordered SAS, as the HeR value measured by wide-line ^1^H NMR indicates. The predicted 3- and 8-state SSs of the stabilin-2 CTD are identical according to both the averaged and the SPIDER3-SPOT-1D methods, except for the prediction of the 8-state averaged results (Figure 3). The stabilin-2 CTD can be described as a uniform coil, except for a short helix motif. The helix can be found near the C-terminal end, at positions 37–41. This is a five residue long motif, and it occupies 10% of the entire length. The predicted 10% is very minute compared with the HeR value, and buried secondary elements are not possible in this case. The sequence-only-based prediction methods are unable to properly handle the stabilin-2 CTD.

The stabilin-2 CTD is predicted to contain fewer secondary structural elements than Tb4, but wide-line ^1^H NMR experimental data prove just the contrary: Tb4 has a more open structure than the stabilin-2 CTD with more binding sites that are free to form a mobile hydration shell.^4,6^

**Figure 4:**
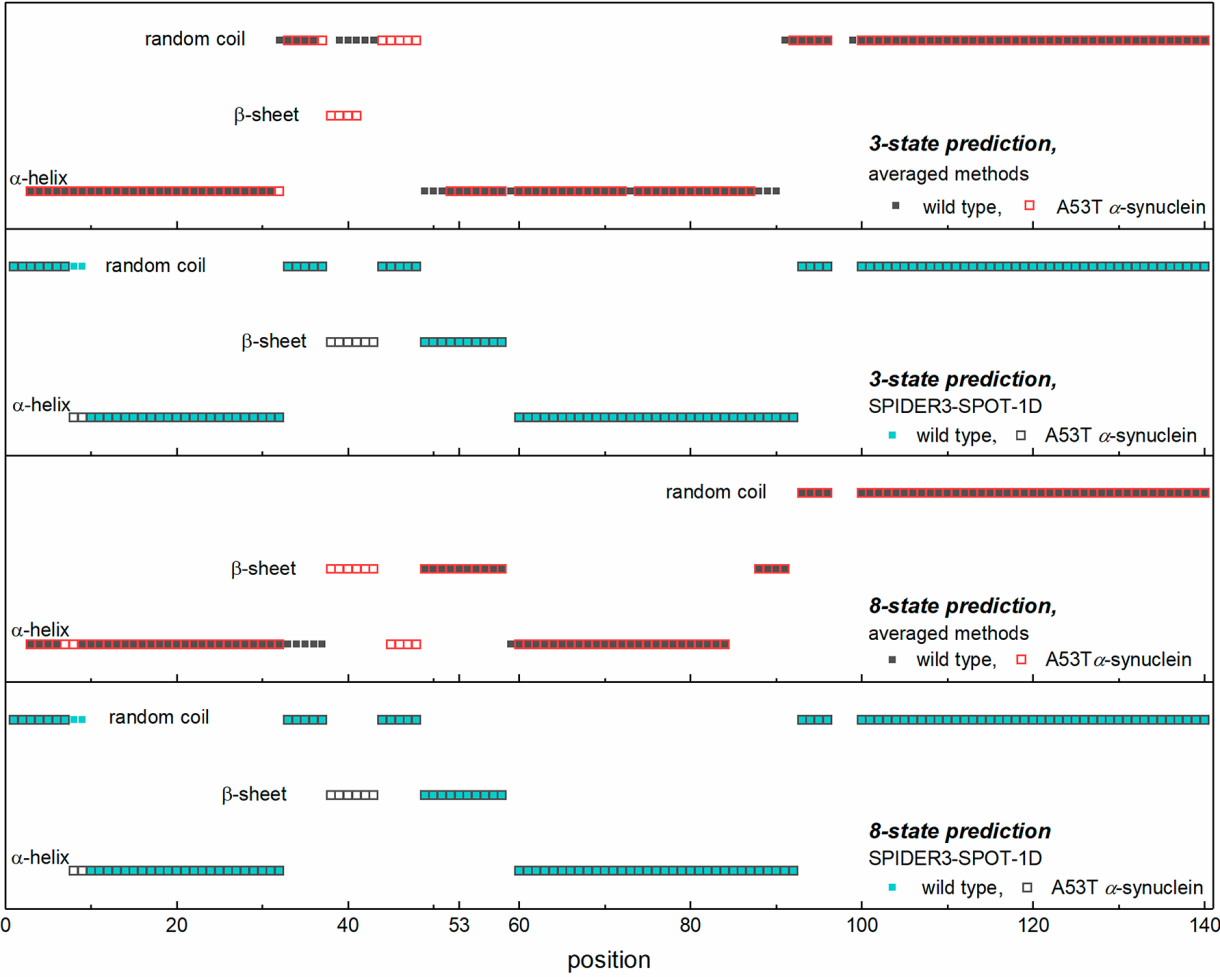
Predicted 3- and 8-state secondary structures as an average structure of five modeling programs (SPIDER3-SPOT-1D, PSRSM, MUFold-SSW, Porter 5, and RaptorX) and the separate SPIDER3-SPOT-1D prediction of secondary structures for wild-type and A53T α-synuclein.

In α-Ss, the determinant motifs are coils, helices, and β-sheets according to 3- and 8-state prediction methods. For WT α-S (Figure 4), the 3-state methods indicate the coil and the helix to be the most determinant. The 8-state SS predicting methods also forecast abundant β-sheets.

The predictions suggest that WT and A53T α-S variants have very similar SSs. The first half of the sequence shows the greatest difference between the two variants. In the results averaged over five 3-state methods, the second short coil region is shifted by five positions toward the C-terminus in A53T relative to WT. A 29 residue long helical section (residues 3–31) can be found in the WT α-S, and a 30 residue long helical section (residues 3–32) can be found in the A53T variant. These helices extend to 21% of the entire protein length. A second helix, in the middle of the proteins, is 42 residues long for the WT (residues 49–90), which is 30% of the protein, whereas it extends to 26% of the protein with a 36 residue length (residues 52–87) for A53T. Together, the two helices add up to 51 (WT) or 47% (A53T) of the entire length according to the average of the five methods.

Helices are responsible for ordered structures, which are detected in 30(4)% of the WT and in 35(4)% of the A53T by wide-line ^1^H NMR (average 32.5(2)%). According to the predictions, the lengths of helices comprise 49% of the whole α-S protein; that is, they are too long compared with the measurements. An explanation of this phenomenon is that parts of the helices are not on the SAS of the protein but are buried in the hydrophobic interior of it. Altogether, helices and β-sheets extend to half of the protein, which considerably overestimates the amount of experimentally determined secondary structural elements.

β-sheets can only be found in the A53T mutant, in the form of a short, four-residue section (residues 38–41), as predicted by averaged 3-state methods. The 3- and 8-state predictions show the appearance of a β-sheet in α-Ss around residue position 40 (Figure 4). The disorder prediction of IUPred2 (Figure 2) indicates greater order in A53T than in the WT α-S at the exact site of the mutation and from it toward the N-terminus. The mutation also entails the increment of β-sheet content that was reported based on experimental results by others.^13−17^

The 3-state prediction of SPIDER3-SPOT-1D (Figure 4) differs significantly in detail from that calculated as an average over five methods for the α-S variants. Coil motifs are shorter by two residues for A53T compared with the WT. SPIDER3-SPOT-1D predicts 21% lower helix content in the WT α-S than the average result. On the contrary, there is a ten-residue difference in A53T; the first coil is longer by two residues, there is an extra β-sheet at positions 38–43, and the first helix is longer by two residues than in WT. There is no β-sheet in the WT variant, and in the A53T mutant it is minimal (four residues at positions 38–41), as predicted by the five averaged 3-state methods. In contrast, the SPIDER3-SPOT-1D method shows 10 (WT: positions 49–58) and 6 + 10 (A53T: positions 38–43 and 49–58) residues to have β-sheet arrangements. In summary, there is a greater β-sheet ratio for the mutant sequence and an excess of β-sheets near the site of mutation or toward the amino terminus, in agreement with all of the above-mentioned predictions.

The 8-state SS prediction methods also show random coils, α-helices, and β-sheets only as the 3-state predictions. The size of the random coils is the same for both the WT and A53T α-Ss (Figure 4) according to averaged predictions. Helices extend to the same number of residues in both the WT and A53T α-Ss in the averaged predictions but at shifted positions and in different sections (WT averaged helices: positions 3–6, 9–37, and 59–84; A53T averaged helices: positions 3–32, 45–48, and 60–84). The β-sheet structure is more extensive in the mutant variant; the 14 residue length in the WT α-S grows to 20 residues in the A53T α-S. An important difference between the averaged and the SPIDER3-SPOT-1D prediction is in the length of the random coil, which is longer by 40% compared with the averaged structure. Moreover, helices and β-sheets are shorter according to the SPIDER3-SPOT-1D prediction.

As a general conclusion of these observations, average of the 8-state predictions forecasts SS for the 55% of the whole protein. SPIDER3-SPOT-1D method, very similarly to this, indicates 50%. The 8-state SS prediction, consequently, overestimates the measured value. The overestimation of structured regions could indicate the predisposition of the disordered monomers to fold upon formation of the amyloid fibrils. The structural traits picked up by the SS predictors mostly remain masked by the high flexibility of the disordered monomers and only become realized when fibril formation occurs.

For α-Ss, the 3- and 8-state methods overestimate the SS content to be ∼50% instead of the measured 35(4)%, as deduced from the HeR parameter of wide-line ^1^H NMR. The ANCHOR2 protein binding region shows the WT and A53T mutant α-Ss to behave identically. The last 35 residues at C-terminus have the possibility of forming bonds.

According to the HeR, the individual Tb4 and the stabilin-2 CTD have very heterogeneous bonds with mobile hydration water molecules. IUPred2 predicts them to be highly disordered in their entire length, in accordance with experimental NMR results.^4^ ANCHOR shows two disordered binding regions of Tb4 and classifies the whole stabilin-2 CTD, so the 3- and 8-state SS predictions of these proteins gave identical results. A helix as large as 23% of Tb4 was predicted, which fit very well the 22(1)% (1–HeR) value, that is, secondary structural elements measured by wide-line NMR.^4^ A smaller helix of 12% is not exposed on the SAS and is not visible for wide-line NMR. The predicted 10% helical content for the stabilin-2 CTD is very minute compared with the HeR value. Tb4 has a more open structure than stabilin-2 CTD, as measured by NMR,^4,6^ in contrast with the predictions.

IUPred2 predicted that both the WT and the a53T α-Ss were partially disordered to 42(8)% (first 80–100 residues) and 82(4)% (last 30 residues), respectively. This agrees with α-Ss being intrinsically disordered. The A53T mutation induces β-sheet formation, but it is not detected by the IUPred2 algorithm, as the single β-strand alone forms a rather extended structure, similar to disordered segments. A stronger β-sheet-forming tendency becomes apparent in the faster fibril formation of the mutant variant, but the mutant appears to be even more disordered than the WT in ^1^H NMR measurements. Despite this, wide-line NMR experiments indicate a more open structure.^5,10^ The IUPred2 prediction indicates a more ordered section in the sequence of the A53T than in the WT α-S. A β-sheet also appears in the 3- and 8-state SS predictions with the A53T mutation. They show an excess of β-sheets in the mutant, indicating its higher capacity to form amyloid fibrils. The determinant motifs are coils, helices, and β-sheets in the α-Ss according to these predictions. They overestimate the SS content compared with the HeR parameter values of wide-line NMR. The WT and A53T mutant α-Ss behave identically, as the ANCHOR2 protein-binding region shows.

## Methods

Protein preparation and wide-line NMR measurements (see the Supporting Information) were described in former publications.^4,5^ Tb4 (44 amino acids, with a starting methionine) and the 2501–2551 amino acid sequence of the stabilin-2 cytoplasmic domain were used for wide-line ^1^H NMR experiments.

The applied 3-state SS prediction methods are SPIDER3-SPOT-1D, PRSM, MUFOLD-SSW, Porter 5, and RaptorX. 8-State predictions were also made with the same methods, except for PRSM.

SPIDER3-SPOT-1D (https://sparks-lab.org/server/spider3/) is a bidirectional recurrent neural network (BRNN) model^18^ that contains long short-term memory (LSTM) cells. The model used in SPOT-1D^19,20^ applies an ensemble of LSTM BRNN and residual convolutional network (ResNet) hybrid models. The method achieves 87 (segment overlap measure (SOV) 80%) and 77% (SOV 75%) in 3- and 8-state SS predictions (Q3 and Q8 accuracy), respectively.^21^ The SPIDER3-SPOT-1D results are also reported individually, not just as included in the average value, because this method gives the most accurate predictions.

PSRSM (http://qilubio.qlu.edu.cn:82/protein_PSRSM/default.aspx) uses methods based on data partitioning and the semirandom subspace method.^22^ In the traditional random subspace method, the low-dimensional subspace is generated by random sampling in a high-dimensional space. First, the training data are divided into different subsets according to the length of the protein sequence; then, the subspace is generated using the semirandom subspace method, and the basic classifier is trained in the subspace. Finally, they are combined by a majority vote rule on each subset. The experiment carried out on six data sets achieves a Q3 result of 85.5% on average (SOV 83.6%)

MUFold-SSW (MUFold Secondary Structure Web server, is a web-based implementation that applies different deep-learning methods and architectures.^23^ The architecture makes possible the effective processing of local and global interactions between amino acid residues and therefore accurate prediction. The accuracy of the method is 85% on level Q3^24^ (82.6% SOV^25^), and it is 74% on level Q8^24^ (71.5% SOV^25^).

Porter 5 (http://distilldeep.ucd.ie/porter/) is composed of ensembles of cascaded BRNNs and CNFs. It incorporates new input encoding techniques and is trained on a large set of protein structures.^26^ Porter 5 achieves 84% accuracy (81% SOV) when tested on three classes and 73% accuracy (70% SOV) when tested on eight classes on a large independent set.

RaptorX Property (http://raptorx.uchicago.edu/) is a web server that predicts the structure properties of a protein sequence without using any templates.^27^ This server employs a powerful in-house deep-learning model, DeepCNF (Deep Convolutional Neural Fields), to predict the SS, solvent accessibility, and disorder regions. DeepCNF not only models the complex sequence–structure relationship by a deep hierarchical architecture but also models the interdependency between adjacent property labels. Experimental results show that this server can obtain ∼84% Q3 (SOV 85%) accuracy for a 3-state SS and ∼72% Q8 (SOV 68%) accuracy for an 8-state SS.

IUPred2A^9,28−30^ was used, which is a combined web interface that allows one to identify disordered protein regions using IUPred2 and disordered binding regions using ANCHOR2.

## Supporting Information Available

Supporting Methods. Figure S1. Melting diagrams of globular proteins. Figure S2. Melting diagrams of intrinsically disordered proteins. Figure S3. Melting diagrams of α-synuclein variants (PDF)
