# Supplementary material for: Secondary Structures of Proteins: A Comparison of Models and Experimental Results
Source: J Proteome Res. 2021 Feb 23;20(3):1802–8. doi: 10.1021/acs.jproteome.0c00986 (PMC8028322; doi:10.1021/acs.jproteome.0c00986)
Supplement: Supplementary file 1 — pr0c00986_si_001.pdf [file pr0c00986_si_001.pdf]

Supporting Information

Secondary structures of proteins, a comparison of models and experimental results

Mónika Bokor, Ágnes Tantos

Table of contents

Supporting Methods   Solid-state-NMR melting diagrams

Figure S1.   Melting diagrams of globular proteins.

Figure S2.   Melting diagrams of intrinsically disordered proteins.

Figure S3.   Melting diagrams of α-synuclein variants.

Supporting Methods

Solid-state-NMR melting diagrams

Melting diagrams of frozen solutions are the relative ratio of mobile water, n represented as a function of normalized functional temperature, T_{fn}. The amount of mobile water can be given as hydration, h also.

Normalized functional temperature, T_{fn} is the absolute temperature normalized to the melting temperature of bulk ice (273.15 K). E_a corresponds to the potential barrier determining the motion of hydration water. It is the normalized functional temperature scaled with the melting heat of ice (6.01 kJ mol^{-1}).

The melting diagrams of globular proteins consist a wide constant region. For more details, see Refs. [1,2] (Fig. S1). The intrinsically disordered proteins have composite melting diagrams [3-7] (Fig. S2). The low-T_{fn} constant region in the melting diagram of intrinsically disordered proteins, if there is any, is originating from secondary protein structures. It means homogeneously bound mobile hydration water molecules. The higher-T_{fn} region with rising mobile hydration values is due to heterogeneously bound mobile hydration water molecules.

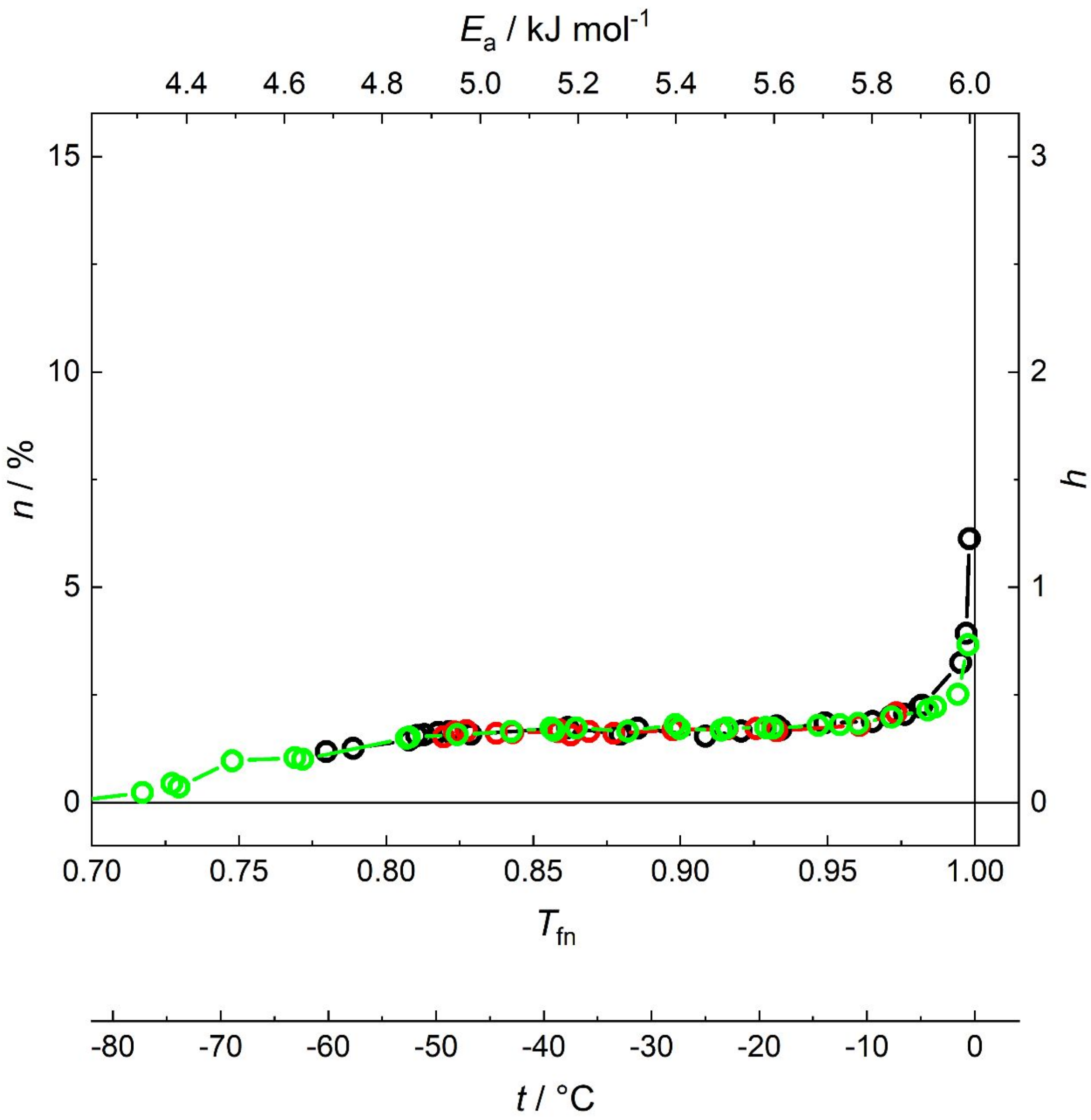

Figure S1. Melting diagrams of globular proteins: bovine serum albumin (black) [1], β-casein (red) [2], and lysozyme (green) dissolved in pure water. n are given for 50 mg/ml concentration.

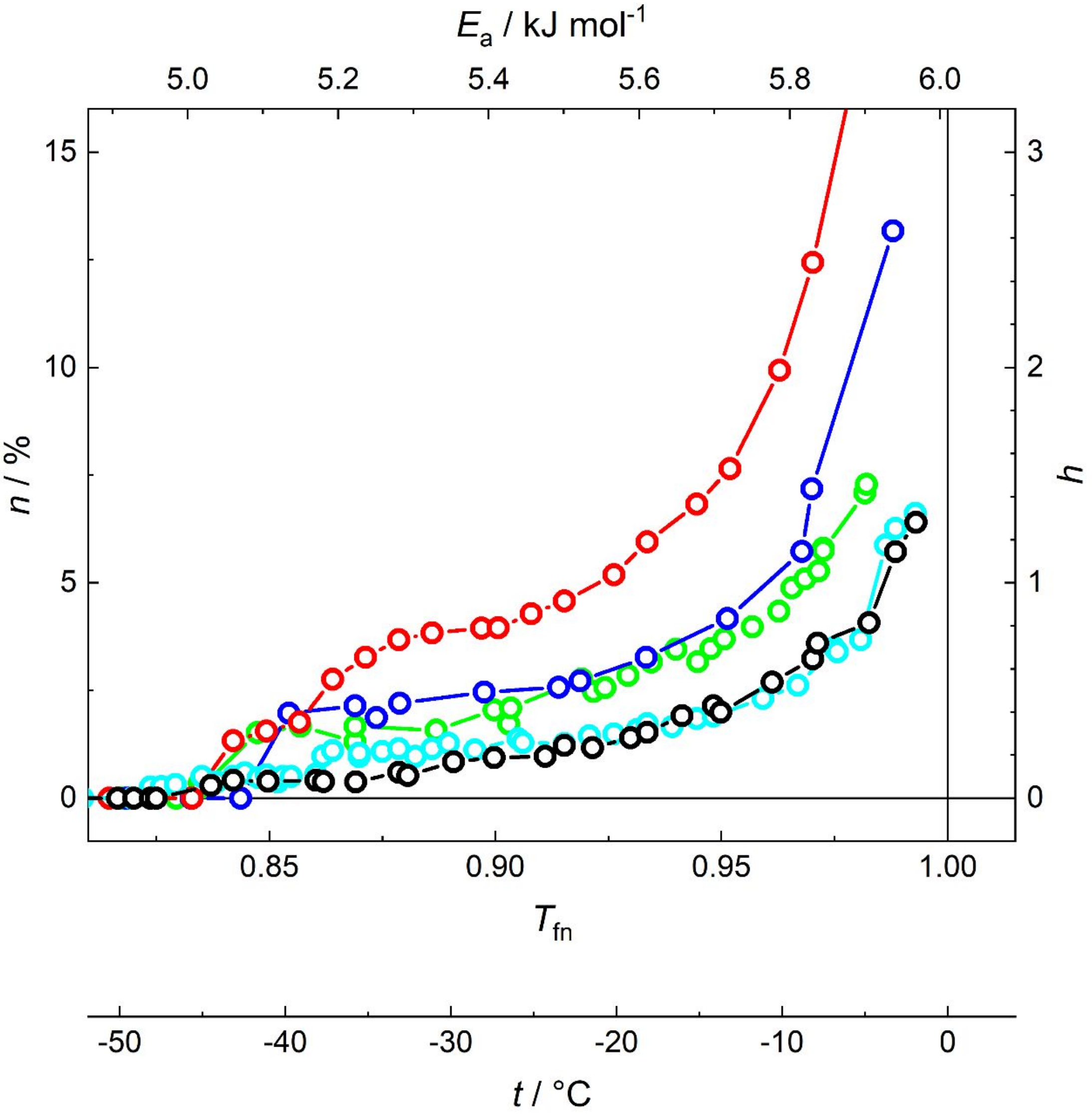

Figure S2. Melting diagrams of intrinsically disordered proteins: cytoplasmic domain of stabilin-2 (black) [3,4], p53 transactivation domain (red) [5], early response to dehydration10 (green) [1], A53T α-synuclein (blue), and thymosin-β_4 (cyan) [6,7]. The proteins are dissolved in pure water. n are given for 50 mg/ml concentration.

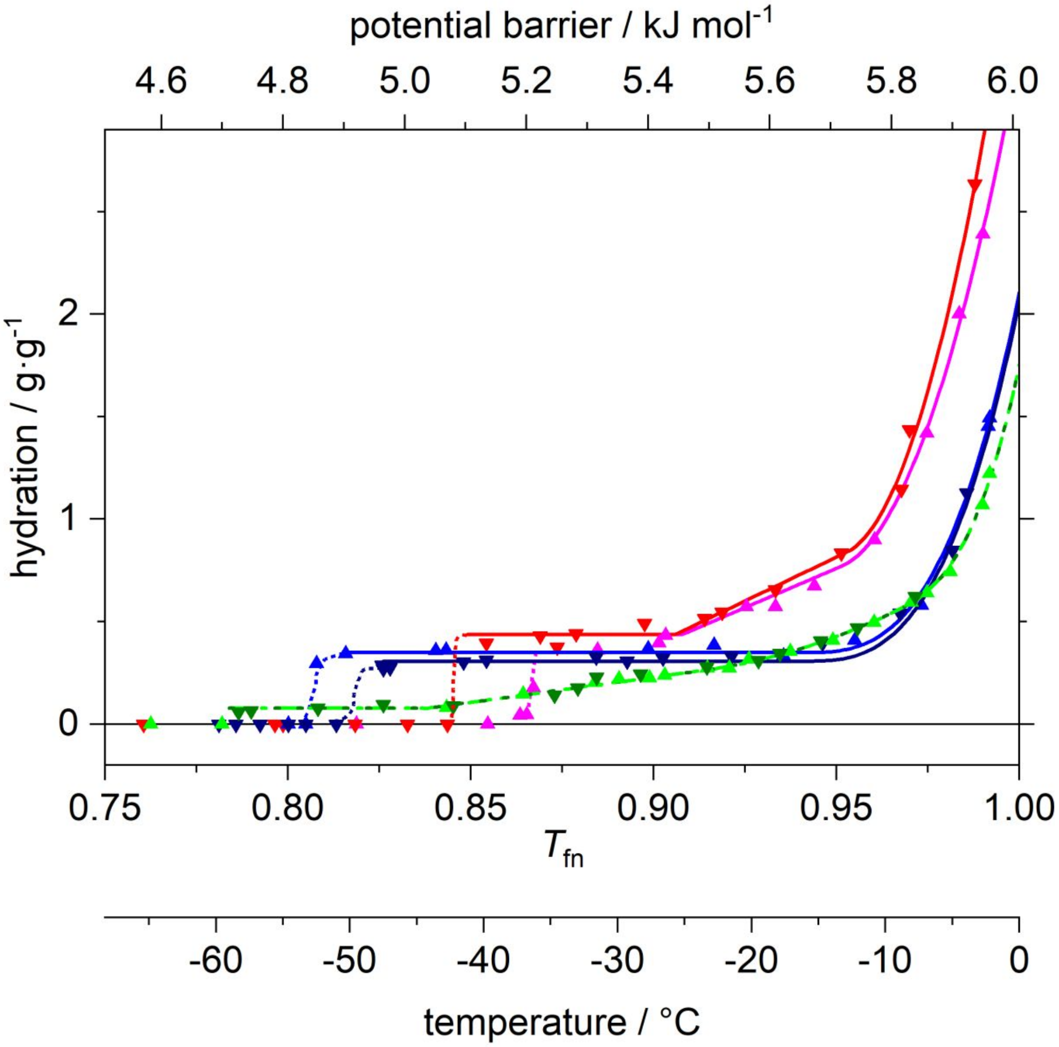

Figure S3. Melting diagrams of α-synuclein variants: wild type (magenta △) and A53T (red ▽) monomers, wild type (blue △) and A53T (dark blue ▽) oligomers, wild type (green △) and A53T (dark green ▽) amyloids.

References

[1] K. Tompa, M. Bokor, P. Tompa. (2018). The Melting Diagram of protein solutions and its thermodynamic interpretation. *Int. J. Mol. Sci.*, **19**, 3571. DOI: 10.3390/ijms19113571

[2] K. Tompa, M. Bokor, P. Tompa. (2019). Globular proteins – melting diagrams of aqueous solutions, thermodynamic interpretation. *Magyar Kémiai Folyóirat*, **125**, 147-155, DOI: 10.24100/MKF.2019.04.147

[3] Á. Tantos, B. Szabo, A. Lang, Z. Varga, M. Tsylonok, M. Bokor, T. Verebelyi, P. Kamasa, K. Tompa, A. Perczel, L. Buday, S. H. Lee, Y. Choo, K.-H. Han, P. Tompa. (2013). Multiple

fuzzy interactions in the moonlighting function of thymosin-β4. *Intrinsically Disordered Proteins*, **1**, e26204, DOI: 10.4161/idp.26204

[4] M. Bokor, Á. Tantos, A. Mészáros, B. Jenei, R. Haminda, P. Tompa, K. Tompa. (2020). Molecular motions and interactions in aqueous solutions of thymosin-β_4, stabilin CTD and their 1:1 complex, studied by ^{1}H NMR spectroscopy. *ChemPhysChem*, **21**, 1420-1428. DOI: 10.1002/cphc.202000264

[5] P. Tompa, K.-H. Han, M. Bokor, P. Kamasa, Á. Tantos, B. Fritz, D.-H. Kim, C. Lee, T. Verebélyi, K. Tompa. (2016). Wide-line NMR and DSC studies on intrinsically disordered p53 transactivation domain and its helically pre-structured segment. *BMB Rep.*, 49, 197-501. DOI: 10.5483/BMBRep.2016.49.9.037

[6] E. Házy, M. Bokor, L. Kalmár, A. Gelencsér, P. Kamasa, K.-H. Han, K. Tompa, P. Tompa. (2011). Distinct hydration properties of wild-type and familial point mutant A53T of α-synuclein associated with Parkinson's disease. *Biophys. J.*, **101**, 2260-2266. DOI: 10.1016/j.bpj.2011.08.052

[7] M. Bokor, Á. Tantos, P. Tompa, K.-H. Han, K. Tompa. (2020). WT and A53T α-synuclein systems: melting diagram and its new interpretation. *Int. J. Mol. Sci.*, **21**, 3997. DOI: 10.3390/ijms21113997
